# Vapor Sublimation and Deposition to Fabricate a Porous Methyl Propiolate-Functionalized Poly-*p*-xylylene Material for Copper-Free Click Chemistry

**DOI:** 10.3390/polym13050786

**Published:** 2021-03-04

**Authors:** Chin-Yun Lee, Shu-Man Hu, Jia-Qi Xiao, Yu-Ming Chang, Tatsuya Kusanagi, Ting-Ying Wu, Ya-Ru Chiu, Yen-Ching Yang, Chao-Wei Huang, Hsien-Yeh Chen

**Affiliations:** 1 Department of Chemical Engineering, National Taiwan University, Taipei 10617, Taiwan; r09524048@ntu.edu.tw (C.-Y.L.); r09524083@ntu.edu.tw (S.-M.H.); r09524013@ntu.edu.tw (J.-Q.X.); r09524079@ntu.edu.tw (Y.-M.C.); r09524111@ntu.edu.tw (T.K.); tilee824@gmail.com (T.-Y.W.); iwbs24921@gmail.com (Y.-R.C.); 2 Department of Tropical Agriculture and International Cooperation, National Pingtung University of Science and Technology, Pingtung 91201, Taiwan; cwhuang@mail.npust.edu.tw; 3 Molecular Imaging Center, National Taiwan University, Taipei 10617, Taiwan; 4 Advanced Research Center for Green Materials Science and Technology, National Taiwan University, Taipei 10617, Taiwan

**Keywords:** vapor deposition, poly-*p*-xylylene, copper-free click chemistry, porous polymer, biointerface

## Abstract

Conventional porous materials are mostly synthesized in solution-based methods involving solvents and initiators, and the functionalization of these porous materials usually requires additional and complex steps. In the current study, a methyl propiolate-functionalized porous poly-*p*-xylylene material was fabricated based on a unique vapor sublimation and deposition process. The process used a water solution and ice as the template with a customizable shape and dimensions, and the conventional chemical vapor deposition (CVD) polymerization of poly-*p*-xylylene on such an ice template formed a three-dimensional, porous poly-*p*-xylylene material with interconnected porous structures. More importantly, the functionality of methyl propiolate was well preserved by using methyl propiolate-substituted [2,2]-paracyclophane during the vapor deposition polymerization process and was installed in one step on the final porous poly-*p*-xylylene products. This functionality exhibited an intact structure and reactivity during the proposed vapor sublimation and deposition process and was proven to have no decomposition or side products after further characterization and conjugation experiments. The electron-withdrawing methyl propiolate group readily provided efficient alkynes as click azide-terminated molecules under copper-free and mild conditions at room temperature and in environmentally friendly solvents, such as water. The resulting methyl propiolate-functionalized porous poly-*p*-xylylene exhibited interface properties with clickable specific covalent attachment toward azide-terminated target molecules, which are widely available for drugs and biomolecules. The fabricated functional porous materials represent an advanced material featuring porous structures, a straightforward synthetic approach, and precise and controlled interface click chemistry, rendering long-term stability and efficacy to conjugate target functionalities that are expected to attract a variety of new applications.

## 1. Introduction

Porous materials have great potential for various applications in drug delivery, heterogeneous catalysis, surface modification, biomedical scaffolds, and so on; these porous materials provide remarkable physical interface properties, such as drug release containers, immobilization of catalysts and molecular sieves [1,2,3], and interconnected pore structures, and render advantages including mass transport, interchange [4], structural morphology and roughness [5]. Promising applications have also been shown in the biomedical engineering field. From the interface chemistry point of view, orthogonal and specific reactions play important roles in ensuring the selectivity of desired reaction routes and preventing unnecessary side products that can precisely target molecules and functions and enable particular reactions efficiently, even in complex and multicomponent environments [6,7,8]. For instance, Staudinger ligation forms amide bonds by the specific conjugation of an azide and a triarylphosphine to produce stable cell-surface adducts [9]. A metal-free click reaction between an azide and a cyclic acetylene was used to specifically bind unnatural sialic acids produced by tetra-acetylated N-azidoacetyl-d-mannosamine on a tumor cell surface [10] and has also reported for dynamic imaging in vivo [11]. A bio-orthogonal reaction was also applied to live cell labeling and showed that tetrazines react irreversibly with the strained dienophile norbornene, forming dihydropyrazine products and dinitrogen [12]. Recent progress by exploiting these click reactions were also demonstrated with success [13,14,15,16]. The development of a porous material that can combine both physical and structural properties and chemical reactivity has been seen sporadically due to the complexity of synthesis, and multiple steps are required for the installation of specific functional conducts [17]. A prospective porous material that comprises both properties is still urgently needed.

**Figure 1:**
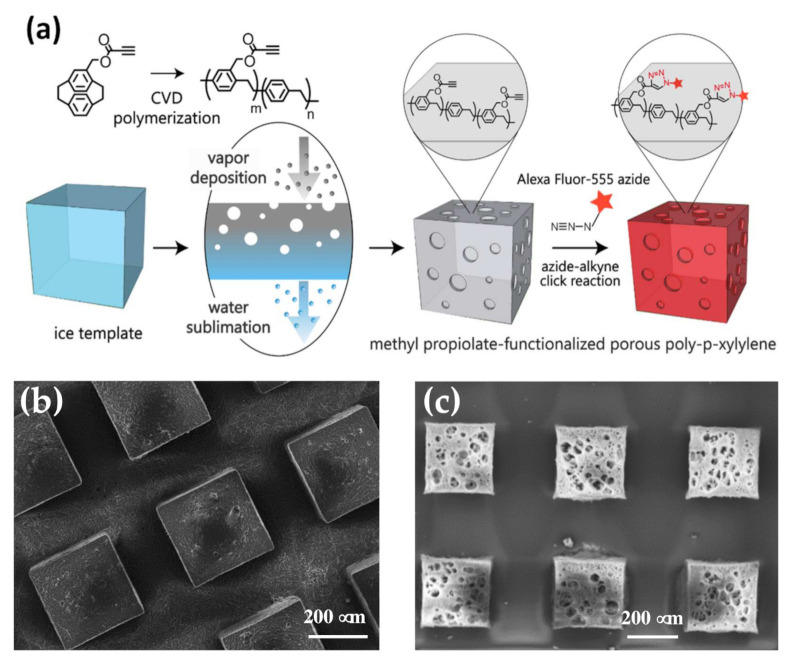
(**a**) Chemical vapor deposition (CVD) polymerization of methyl propiolate-substituted [2,2]-paracyclophane to form a poly((methyl propiolate-*p*-xylylene)-*co*-(*p*-xylylene)) porous structure and schematic illustration of vapor sublimation and deposition to fabricate a three-dimensional and porous structure that can undergo a copper-free click reaction with azide-terminated molecules. (**b**) SEM micrographs of the fabricated ice templates and (**c**) the resultant porous poly((methyl propiolate-*p*-xylylene)-*co*-(*p*-xylylene)) by the proposed fabrication process. An array structure composed of 300 μm × 300 μm × 300 μm cubes was used in the study for demonstration.

We therefore introduced a vapor-based synthesis of a porous material that comprised poly-*p*-xylylene as the backbone matrix and was equipped with methyl propiolate functionality to perform a metal-free 1,3-dipolar cycloaddition click reaction from the interface. The construction of the porous material is based on our previous discovery that the deposition of poly-*p*-xylylene on sublimated ice templates (instead of non-sublimated substrates) can result in the production of a three-dimensional and porous poly-*p*-xylylene material [18], and the shape, size, and inner porous structure of the porous polymer products are also customizable [19,20]. It has also been reported that the deposition of poly-*p*-xylylenes can be functionalized by using substituted [2,2]-paracyclophanes as the precursors during the chemical vapor deposition (CVD) polymerization process [21], and a variety of functional groups, such as amine, hydroxyl, vinyl, alkyne, maleimide, aldehyde, and disulfide groups, which were successfully produced to form functionalized poly-*p*-xylylene coatings and performed tasks for biointerface engineering [22,23,24,25,26,27,28,29]. Based on previous findings, we hypothesized that vapor deposition from methyl propiolate-substituted [2,2]-paracyclophane on a sublimated ice template can result in the formation of a porous and methyl propiolate-functionalized poly-*p*-xylylene material during one vapor sublimation and deposition step, and a schematic illustration of this process is shown in Figure 1a. The resulting functional and porous material product exhibited (i) a porous property of interconnected pore structures within the polymer network and (ii) an interface property of functionalized methyl propiolate conducts that are able to perform a site-specific and metal-free click reaction. The vapor fabrication process is a dry and clean process that is free of chemical solvent, catalysts, and initiators, and the product polymer material belongs to the poly-*p*-xylylene family as classified by the United States Pharmacopoeia (USP) as the highest-Class VI biocompatible materials representing an excellent candidate for biotechnological applications. The introduced fabrication method and polymer product represent a standard example of a biointerface material that provides both physical porous properties and chemical interface properties for emerging sophisticated biomedical applications.

## 2. Materials and Methods

### 2.1. Fabrication Process

Ice templates with dimensions of 300 µm × 300 µm × 300 µm were prepared for the fabrication of poly-*p*-xylylene porous materials via the reported vapor sublimation and deposition process. By using a customized negative mold of polydimethylsiloxane (PDMS), which was created following a previously reported method [30], deionized water was then filled inside the mold. After freezing in a bath of liquid nitrogen or dry ice, the ice templates with the desired dimensions were completed and readily used in the vapor sublimation and deposition process. For vapor deposition, self-built deposition equipment was used in the current study, and the starting materials of [2,2]-paracyclophane was obtained commercially (Galentis, Marcon, Italy), while the methyl propiolate-[2,2]-paracyclophane was synthesized following the procedures reported elsewhere [26,31]. Briefly, these starting materials were first vaporized under a reduced pressure of 75 mTorr and at a temperature of approximately 100 °C, followed by high-temperature pyrolysis at approximately 670 °C for [2,2]-paracyclophane and 510 °C for methyl propiolate-[2,2]-paracyclophane, and the vaporized starting materials were transformed into the corresponding *p*-xylylene (*p*-quinodimethane), which is a reactive monomer for further polymerization and deposition. The ice templates (vapor sublimation) prepared beforehand were placed in a deposition chamber awaiting the monomers (vapor deposition) polymerized on them to fabricate the final poly-*p*-xylylene porous material. In addition, the carrier gas of argon with a mass flow rate of 25 sccm was applied to transfer both the vaporized starting materials ([2,2]-paracyclophanes) and the transformed *p*-xylylenes during the whole process. The deposition rate was regulated at approximately 0.5 Å/s to 1.0 Å/s based on the feeding amount of the starting materials and was monitored by using a real-time quartz crystal microbalance (QCM) sensor (STM-100/MF, Sycon Instruments, East Syracuse, NY, USA) that was installed at the deposition chamber.

### 2.2. Characterizations

Cryo-SEM (Tabletop TM-3000, Hitachi, Tokyo, Japan) was used to image the ice template, which was kept cooled by continuously supplied liquid nitrogen. The cryo-SEM was operated with an electron energy of 15 keV and a pressure of 100 mTorr. The resultant poly-*p*-xylylene porous cubes were analyzed by Nova NanoSEM 230 SEM (FEI, Hillsboro, Oregon, USA), which was operated under a reduced pressure of 4 × 10^−6^ Torr and at room temperature. Three-dimensional images of the interior porous structure were recorded using a micro-CT X-ray imaging system (SkyScan 1176, Bruker, Billerica, MA, USA). The samples were scanned at a voltage of 40 kV, the scanning resolution was 9 µm voxels with an integration time of 2000 ms for each projection, and the conversion of the collected projection images into a 3-D image was performed by CTvox software (Bruker, Billerica, MA, USA). Real-time mass spectrometry analysis was performed using a residual gas analyzer (RGA, Hiden Analytical, Warrington, UK) that was mounted on the deposition chamber and operated at an ultrahigh vacuum of 10^−9^ Torr with an ionization electron energy of 70 eV and ionization emission current of 20 µA. A Spectrum 100 FT-IR spectrometer (PerkinElmer, Waltham, Massachusetts, USA) equipped with a liquid nitrogen-cooled mercury–cadmium–telluride (MCT) detector and an advanced grazing angle specular reflectance accessory (PIKE Technologies, Fitchburg, WI, USA) was used for the surface characterization of the material. The scan range was from 500 cm^−1^ to 4000 cm^−1^, and 64 scans were performed for each acquisition. XPS (X-ray photoelectron spectroscopy) measurements were carried out by a Theta Probe X-ray photoelectron spectrometer (Thermo Scientific, Leicestershire, UK) using monochromatized AlKα radiation as the X-ray source with a power of 150 kW. Under a pass energy of 20 eV, high-resolution C_1s_ elemental analysis was performed, and the experimental results were compared to the theoretical values calculated on the basis of the structures.

### 2.3. Conjugations

The conjugation of the Alexa Fluor^®^ 555-labeled azide (Thermo Fisher Scientific, Waltham, Massachusetts, USA) was performed by reacting a 5 mM solution of the molecule with the methyl propiolate groups of the fabricated porous material samples at 20 °C for 4 h. Control experiments by reacting the same Alexa Fluor^®^ 555-labeled azide agent to a non-functionalized poly-*p*-xylylene was performed in parallel for the comparison. A wash process was performed to remove excess and unreacted reagents for both of the conjugated samples and the samples from the control experiments with the following procedures: three times with PBS (pH = 7.4, containing Tween 20, Sigma-Aldrich, St. Louis, MI, USA), one time with PBS (without Tween 20) and finally a deionized water rinse. Fluorescence images were recorded and collected with a TCS SP5 CLSM confocal laser scanning microscope (Leica Microsystems, Wetzlar, Germany), and the light source was a HeNe laser (wavelength: 532 nm) for the detection of the Alexa Fluor^®^ 555-labeled azide (emission wavelength: 580–600 nm).

## 3. Results and Discussion

Fabrication of the propiolate-functionalized poly-*p*-xylylene porous materials started by preparing an ice template with selected shapes and dimensions by using a customized PDMS (polydimethylsiloxane) mold, and an array structure composed of 300 μm × 300 μm × 300 μm cubes was used in this study for demonstration. Intuitively, molded and solidified ice cubes were fabricated as ice cubes with larger dimensions that are normally seen in daily life, and the results are shown in the cryo-SEM (scanning electron microscopy) images in Figure 1b. On the other hand, the starting material 4-methyl propiolate-[2,2]-paracyclophane was synthesized following procedures reported elsewhere [31]. Based on the refined Gorham process [32] of chemical vapor deposition (CVD) polymerization, the 4-methyl propiolate-[2,2]-paracyclophane sublimated at approximately 100 °C and was pyrolyzed at 670 °C, forming 4-methyl propiolate-quinodimethanes (monomer), and these monomers could finally polymerize on a cooled surface at room temperature (or below), forming 4-methyl propiolate-poly-*p*-xylylene thin films [26,31], where the entire CVD process occurred at a reduced pressure of 150 mTorr. Herein, in this study vapor deposition was additionally applied on a sublimating ice template, and polymerization occurred on a dynamic interface of a sublimated ice surface instead of the conventionally used static solid surface. Once the ice templates and the starting materials were readily available, commencement of the fabrication was performed based on the reported vapor sublimation and deposition process [18,20] under devised thermodynamic conditions at approximately 15 °C and 150 mTorr. More specifically, under such conditions, the ice template sublimated from its solid phase to the vapor phase (vapor sublimation), while simultaneously, under the same conditions, 4-methyl propiolate-[2,2]-paracyclophane favored vapor deposition and polymerized to form poly-*p*-xylylene. These two processes occurred by consuming the same volume space as that of the ice template but exhibited opposite mass flux directions with a controllable mass balance by sublimation rate and deposition rate [18]. An RGA (residual gas analyzer) was used in the CVD process while the whole system was under a reduced pressure environment to monitor gas composition in real time.

**Figure 2:**
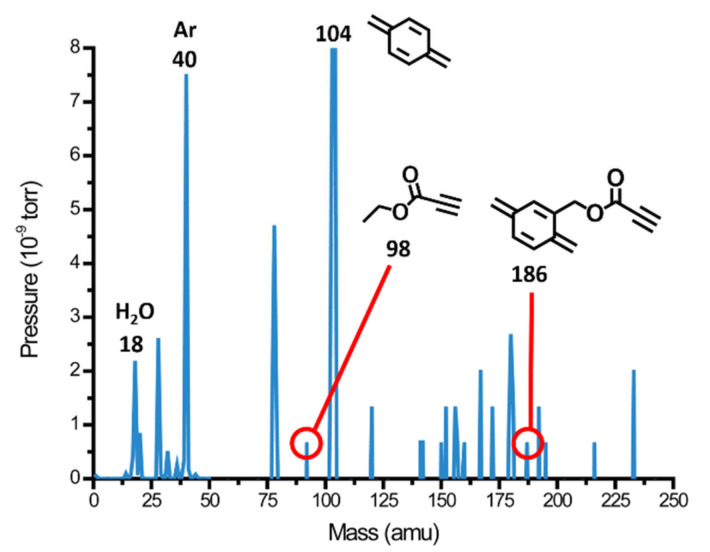
Analysis of vapor compositions during the vapor sublimation and deposition fabrication process. The residual gas analyzer (RGA) results showed the expected presence of sublimating water molecules at 18 amu and the depositing molecules that were composed of methyl propiolate at 98 amu, quinodimethane at 104 amu, and methyl propiolate-substituted quinodimethane at 186 amu. The carrier gas of argon at 40 amu was also detected.

**Figure 3:**
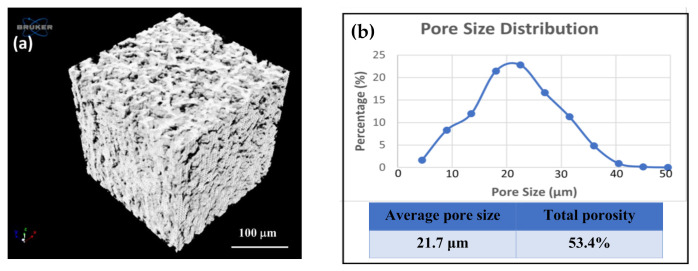
Analysis of porous structure and porosity. (**a**) Three-dimensional and compiled micro-CT images show the porous structure of the fabricated methyl propiolate-functionalized poly-*p*-xylylene. (**b**) Pore size distribution based on micro-CT analysis showing an average pore size of 21.7 μm and a total porosity of 53.4%.

Figure 2 shows the results of the RGA in which the mass number of 18 amu represented the sublimating water vapor in the background with the additional mass number of 40 amu being the carrier gas argon. Furthermore, the deposition components included methyl propiolate, quinodimethane (activated *p*-xylylene) and methyl propiolate-substituted quinodimethane, which were also detected as the mass numbers at 98 amu, 104 amu, and 186 amu. Therefore, the results indicated that the starting material, functionalized [2,2]-paracyclophane, was sublimated and pyrolyzed successfully into reactive monomers in the process and could be transferred to an ice template for subsequent deposition. The ultimate results of the two processes rendered the construction of a three-dimensional porous material of propiolate-functionalized poly-*p*-xylylene with controllable pore size and porosity, and the outlook architecture in terms of shape and size dimensions replicated the ice template. Similar to other porous poly-*p*-xylylene reported [33], pore sizes ranging from 18.7 μm ± 6.7 μm to 35.6 μm ± 8.2 μm and porosities of approximately 54.3–67.6% were measured based on microscopic, porosimetry, and gas adsorption techniques. The resultant porous constructs were also visualized by SEM (scanning electron microscopy), as shown in Figure 1c, where porous cubes of propiolate-functionalized poly-*p*-xylylene with the same dimensions (300 μm × 300 μm × 300 μm) were observed to be consistent with the ice templates, and an average pore size of 25.9 μm ± 9.3 μm was measured. Moreover, micro-CT (micro computed tomography) was applied in the measurement of the porous poly-*p*-xylylene material, as shown in the image in Figure 3a. Micro-CT images clearly illustrated the three-dimensional and homogeneous porous structure of the resulting poly-*p*-xylylene after fabrication. Because of vapor sublimation and deposition during the CVD process, the vapor had to be continuously drilled out of the cubes, causing almost all pores to be open and interconnected with each other. Additionally, an obvious cube appearance provided evidence that the patterns were maintained well compared to the ice template beforehand. In addition, in Figure 3b, the pore size distribution presented a Gaussian distribution that was plotted ranging from 5 μm to 50 μm and mainly focused at approximately 20 μm. It was recorded with an average pore size of 21.7 μm and a voidage of 53.4%. These results showed that the porous structure can provide a large surface area compared to the conventional thin film and possesses greater potential in various applications.

**Figure 4:**
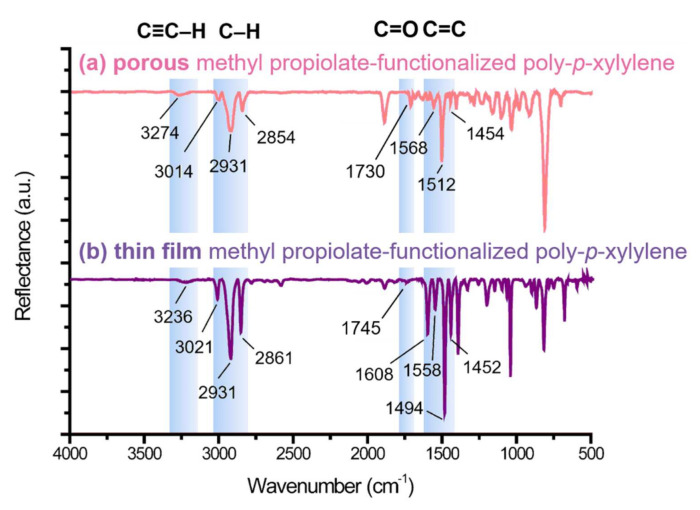
FT-IR spectra showing the comparison of (**a**) the porous methyl propiolate-functionalized poly-*p*-xylylene fabricated from an ice template and (**b**) the thin film methyl propiolate-functionalized poly-*p*-xylylene that was deposited on a conventional gold-coated silicon wafer solid surface. The characteristic bands were found to be consistent in both (**a**,**b**) to confirm the chemical structure.

**Figure 5:**
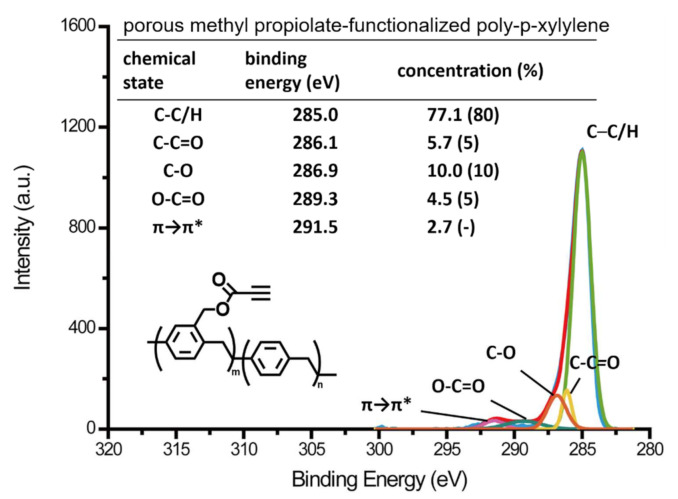
The XPS high-resolution C_1s_ spectra confirmed the details of the chemical structure of the fabricated porous methyl propiolate-functionalized poly-*p*-xylylene. The experimental values of the binding energies and the component concentrations were found to be consistent with the calculated theoretical values.

The resulting poly-*p*-xylylene was then characterized using a combination of FT-IR (Fourier transform infrared spectroscopy) and XPS (X-ray photoelectron spectroscopy). The FT-IR spectra are shown in Figure 4 to determine the surface chemical structure of the material. The spectrum in Figure 4a indicates porous poly-*p*-xylylene in the current study and was compared to the spectrum (Figure 4b) of conventional poly-*p*-xylylene thin films reported in previous research [26]. The bands and positions were closely consistent for both the porous and thin film samples, of which the significant characteristic bands at 3274 cm^−1^ and 3236 cm^−1^ were attributed to the terminal alkyne groups at the side chain, while the characteristic *-C-H* bands were detected in the wavenumber region of 3021 cm^−1^–2854 cm^−1^. In addition, alkene groups were recorded in the wavenumber region from 1608 cm^−1^ to 1452 cm^−1^, and the bands at 1730 cm^−1^ and 1745 cm^−1^ in the spectra were indicative of the carbonyl in ester, which exists at the side chain. With the data comparing the two groups of samples, it was proven that methyl propiolate-functionalized poly-*p*-xylylene in the form of a porous material was successfully synthesized via vapor sublimation and deposition and contributed the same chemical structure when compared to a conventional thin film coating of the same polymer. The XPS analysis, on the other hand, shown in Figure 5 confirms the chemical composition of the porous methyl propiolate poly-*p*-xylylene. The high-resolution C_1s_ spectra revealed that the characteristic signal of aliphatic and aromatic carbon (C-C, C-H) exhibited a signal at 285.0 eV, carbon in the α-position to the carbonyl group (C-C = O) was at 286.1 eV, and C-O and O-C = O bonds were at 286.9 eV and 289.3 eV, respectively. Moreover, *C-C* and C-H bonds exhibited an experimental intensity of 77.1 atom%, which was in accordance with the theoretical concentration of 80 atom%. For C-C = O, C-O and O-C = O bonds, the exact intensities were 5.7 atom%, 10.0 atom%, and 4.5 atom%, respectively, which also matched well with the theoretical values (5 atom%, 10 atom%, and 5 atom%). The signal at 291.5 eV can be attributed to π–π*, which is characteristic of π electrons in aromatic molecules and has been previously reported for other poly-*p*-xylylenes [22,25,28,29]. In summary, with the results from the combination of FT-IR and XPS analysis, it was confirmed that the porous methyl propiolate poly-*p*-xylylene can be successfully synthesized and that no side reactions or decomposition were observed during the CVD polymerization process.

**Figure 6:**
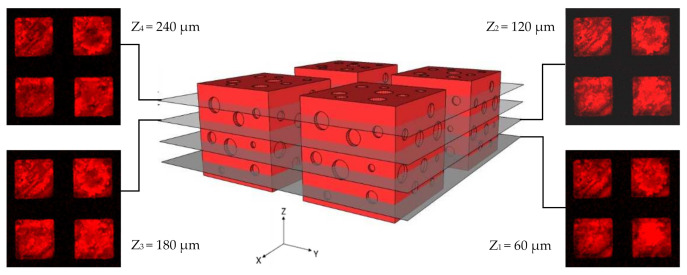
The fabricated porous methyl propiolate-functionalized poly-*p*-xylylene exhibited interface chemistry that can undergo a copper-free azide-alkyne click reaction by the conjugation of Alexa Fluor-555^®^ azide to the equipped alkyne conductor. CLSM images recorded in varied positions along the *Z*-axis show the fluorescence signals at the interfaces of the three-dimensional structure and confirmed that the biorthogonal click reaction was performed. Scale bars are 200 μm.

Importantly, verification of the reactivity of the modified methyl propiolate groups to click alkynes toward an azide functionality was further examined. Compared to ring-strained cyclooctyne [11,34], which was challenging to synthesize, the electron-withdrawing methyl propiolate group used herein provided advantages that were straightforward to synthesize, containing efficient alkyne ready to click azide-terminated molecules under copper-free conditions at room temperature and in friendly solvents, such as water, the functionality was also well preserved during the proposed vapor sublimation and deposition process and was proven with no decomposition or side products by the aforementioned characterizations. The resultant methyl propiolate-functionalized porous material exhibited interface properties with a click reaction of specific covalent attachment of the target molecules, long-term stability and efficacy, and precisely controlled interface chemistry compared to other physical approaches in which poorly controlled adsorption and desorption issues often occurred [35]. During the verification experiments, azide-terminated Alexa Fluor-555^®^ was selected to react with the fabricated methyl propiolate-functionalized porous material, and the reaction was carried out in a water solution at 20 °C without using the copper catalyst. The fluorescence signals in the red channel were observed by using confocal laser scanning microscopy, and as indicated in the micrographs in Figure 6, the fluorescence signals appeared in confined areas with consistent dimensions (300 μm × 300 μm) in the collected 2-D images, corresponding to the fabricated cubes of the methyl propiolate-functionalized porous materials. A thorough washing experiment was conducted for the conjugated samples to confirm the covalent attachment of the azide-Alexa Fluor-555^®^ molecules, and the fluorescence signals before and after washing were found with consistent intensities within the margin of error. A separate control experiment was also performed based on using an unsubstituted [2,2]-paracyclophane to fabricate nonfunctionalized porous poly-*p*-xylylene samples (via the same vapor sublimation and deposition process); the resulting control samples showed suppressed fluorescence signals when applying the same azide-Alexa Fluor-555^®^ molecules, but the loosely bound molecules were then washed away during the wash experiments. Such a cross-reaction control experiment was also reported to be useful in other poly-*p*-xylylene systems to confirm specific reactions [24,36]. In addition, fluorescence images were recorded in different focal planes along the *Z*-axis with a devised interval spacing of 60 μm, i.e., Z-positions were examined at Z_1_ = 60 μm, Z_2_ = 120 μm, Z_3_ = 180 μm, and Z_4_ = 240 μm of a total height of 300 μm, and these images were displayed separately instead of showing a reconstructed 3-D image to better convey the important interface information, which might be concealed due to overlapped fluorescence signals from each focal plane image. The fluorescence images in Figure 6 collectively show distinct conjugation patterns, revealing that the conjugation of azide-Alexa Fluor-555^®^ was attached to the interface of the methyl propiolate-poly-*p*-xylylene porous structure and that the reaction covered the entire porous sample. With respect to the discovered pore size of 25.9 μm, no diffusion limitation for the azide-Alexa Fluor-555^®^ molecules in-and-out of the structure can also support the thorough reaction of the porous samples. With also previously reported successes of using methyl propiolate-poly-*p*-xylylene thin films to conjugate azide-terminal molecules including fluorescently-labeled azides [37], azide-RGD [24], azide-PEG [26], azide-PEG-NHS ester linker with subsequently assembled growth factor proteins [38], a wide spectrum of extended applications is expected. Successful fabrication of methyl propiolate-poly-*p*-xylylene porous material and the installed methyl propiolate being well-preserved functional to perform copper-free click reaction to attach azide molecules were proven useful to perform specific conjugation of targeting molecules and applications.

## 4. Conclusions

A dry and clean vapor sublimation and deposition process was shown to successfully fabricate the porous material of functionalized poly-*p*-xylylene. Moreover, the installed functionality of methyl propiolate was able to perform a site-specific and metal-free click reaction to target an azide-terminated biomolecule. Advantages were found for the introduced fabrication process and the resultant polymer product: an agreeable and friendly process was exploited, the product belonging to the highly biocompatible poly-*p*-xylylene family, with enhanced biointerface properties in which both physical porous property and chemical reactivity were simultaneously creating this material. Applications of using such an advanced material shall not limit but be extendable to the current reported works on the specific immobilization of growth factor proteins, sensing molecules, functional agents, functional monomers and polymers. We foresee prospect applications in tissue engineering, regenerative medicine, targeted drug-eluting materials, and multifunctional imaging materials fields.

## Data Availability

The data presented in this study are available on request from the corresponding author.
